# Development of fricative sound perception in Korean infants: The role of language experience and infants’ initial sensitivity

**DOI:** 10.1371/journal.pone.0199045

**Published:** 2018-06-13

**Authors:** Minha Shin, Youngon Choi, Reiko Mazuka

**Affiliations:** 1 Department of Psychology, Chung-Ang University, Seoul, South Korea; 2 Laboratory for Language Development, RIKEN Center for Brain Science, Tokyo, Japan; 3 Department of Psychology and Neuroscience, Duke University, Durham, NC, United States of America; Utrecht University, NETHERLANDS

## Abstract

In this paper, we report data on the development of Korean infants’ perception of a rare fricative phoneme distinction. Korean fricative consonants have received much interest in the linguistic community due to the language’s distinct categorization of sounds. Unlike many fricative contrasts utilized in most of the world’s languages, Korean fricatives (/s*/-/s/) are all voiceless. Moreover, compared with other sound categories, fricatives have received very little attention in the speech perception development field and no studies thus far have examined Korean infants’ development of native phonology in this domain. Using a visual habituation paradigm, we tested 4‒6-month-old and 7‒9-month-old Korean infants on their abilities to discriminate the Korean fricative pair in the [a] vowel context, /s*a/-/sa/, which can be distinguished based on acoustic cues, such as the durations of aspiration and frication noise. Korean infants older than 7 months were able to reliably discriminate the fricative pair but younger infants did not show clear signs of such discrimination. These results add to the growing evidence that there are native sound contrasts infants cannot discriminate early on without a certain amount of language exposure, providing further data to help delineate the specific nature of early perceptual capacity.

## Introduction

Past research on the development of speech perception suggests that infants’ perception goes through a reorganization during the first year of their lives. In the most dominant pattern, infants discriminate non-native as well as native phonetic contrasts early on but become attuned to the sounds of their native language by the end of the first year. This pattern is referred to as the perceptual narrowing pattern [1–3]. The fact that this was the most frequently documented pattern has played a key role in shaping theories such as the Perceptual Assimilation Model [4–6], the Native Language Magnet Model [7], and the Processing Rich Information from Multidimensional Interactive Representations framework [8] to explain the mechanisms underlying this developmental change.

However, a growing number of studies have shown that the development of infants’ sensitivity to some phonetic contrasts follows different patterns [9–13]. For example, young Filipino infants do not show an ability to discriminate between the nasal sound contrast [na] and [ŋa] early on, but they are able to distinguish them by 10–12 months of age [12]. Japanese infants showed a similar pattern in their development of long/short vowel discrimination [11] and singleton-geminate stop contrast (/pata/ vs. /patta/) [10]. These studies show poor discrimination abilities at a younger age, but as infants grow, they develop the sensitivity needed to discriminate between the sound contrasts. We call this pattern of development the “enhancement” pattern [14]. These findings also suggest that developmental patterns may diverge depending on the acoustic parameters that distinguish specific contrasts. Identifying phonetic contrasts that are difficult for young infants to discriminate, especially those in their native language, can illuminate the bounds of the infants’ early sensitivity in segmental discrimination. It can also elucidate the process by which infants gain the sensitivity required to be able to discriminate those contrast with maturation and increasing language experience.

In the field of infant speech perception development, a limited number of contrasts in vowels and consonants have been experimentally examined. Among these, the voicing contrasts of stops are one of the most extensively tested contrasts to date [15–19]. By comparison, fricatives have received very little attention. As discussed further below, a few early studies of English-learning infants’ discrimination of voicing contrasts in fricatives [20–22] reported that 1‒3-month-old infants could not discriminate the fricative voicing contrast, as in [sa]-[za], and it was not until 6‒8 months that they became able to do so. This is in contrast to the voicing contrast in stops, which English-learning infants were found to discriminate as young as 1 month of age [15]. Unfortunately, these earlier results have not received much attention since they were considered as exceptions due to their methodological limitations. A more recent study by Best and McRoberts [5] tested American infants on English alveolar fricatives and isiZulu lateral fricatives. However, as their study did not include infants younger than 6 months, it is not clear whether infants younger than 6 months would have been able to discriminate these contrasts.

The present study attempts to re-open this unexplored area by testing Korean infants’ ability to discriminate a fricative contrast in their native language. In particular, we examined whether young infants can distinguish the contrast and, if not, when such ability develops, by testing Korean infants from 4 to 9 months of age.

Korean utilizes a rare type of fricative contrast. In many European languages, fricatives contrast both in voicing and in place of articulation. In English, for example, voiced and voiceless fricatives occur at different places of articulation, such as [v]-[f] at the lips, [z]-[s] at the alveolar ridge, and [ð]-[θ] pairs at the interdental place (see also the case of isiZulu, 5). The difference in aspiration duration related to voice onset time (VOT) is known to serve as a main cue in distinguishing these categories [23–25]. In Korean, the only fricative contrast is between fortis /s*/ and lenis /s/, both of which are voiceless. An example of a minimal pair is [s*al] ‘rice’ versus [sal] ‘flesh’ [26]. It should be noted that Korean has a three-way contrast in stops and affricates, all of which are voiceless: fortis, lenis, and aspirated. Note also that Korean stops occur at three places of articulation: labial, alveolar, and velar [27].

While both fortis and lenis fricatives fall in the positive range of VOT spectrum, the VOTs for the fortis category tend to be shorter than those for the lenis category [23–25]. In addition to aspiration duration, frication noise duration plays a key role in the discrimination of fricative sounds, owing to its manner of articulation (air turbulence caused by partial blockage of air flow at each place of articulation) [23–25]. Recent studies have proposed that the fundamental frequency (f0) of the vowels following the fricative consonant and the amplitude difference between the first and second harmonics (H1−H2) can play additional roles in the distinction of Korean fricative [s*] versus [s] [24, 25, 28]. English fricatives have also been found to show significant differences in frication noise duration, aspiration duration, H1−H2, and f0 [29].

Using a high-amplitude sucking procedure, Eilers and Minifie [20] observed that 1‒3-month-old English-learning infants could distinguish fricative contrasts that differ in the place of articulation, such as /sa/-/va/ and /sa/-/ʃa/. However, they did not show clear signs of discrimination for the voicing contrast /sa/-/za/. Later, using a conditioned head-turn method, Eilers, Wilson, and Moore [21] further demonstrated that /sa/-/za/ discrimination is developed around 6‒8 months after birth, and for another fricative pair, /f/-/θ/, the ability to discriminate the contrast may not emerge until 8 months of age. These results seem to suggest that infants’ ability to discriminate the voicing contrast in fricatives may follow an enhancement pattern, rather than the typical perceptual narrowing pattern. However, these results are only partially informative because the method used to test the younger group in [20] differs from that in [21] and [5], disallowing direct comparisons across age groups.

In the present study, we tested Korean infants on their ability to discriminate the [s*]-[s] (fortis-lenis) contrast across two age groups, 4‒6-month-olds and 7‒9-month-olds, using a well-established and widely-accepted infant-testing procedure (that is, the visual habituation method [30]) in our study. The goals of the present study are two-fold. First, we will examine whether fricative voicing contrasts are generally difficult for young infants, such that Korean-learning infants, like their English-learning counterparts, are unable to discriminate them at a young age and only become able to do so as they grow older–that is, develop in the enhancement pattern. Given that every study that tested infants’ discrimination of fricative contrasts was carried out with English-learning infants, evidence from Korean, which is not only typologically very different from English but also has a rare fricative contrast, can add valuable data to the literature.

Second, we will also examine whether infants’ sensitivity to the fricative contrasts differs when the two categories are both in the voiceless dimension. Among stop contrasts, very young infants were found to be sensitive to stops that cross a +30ms VOT boundary (i.e., the English and German type), but only older infants were able to discriminate French [18] and Spanish [31] stop contrasts, whose boundaries are near 0ms VOT. Since we know very little regarding infants’ sensitivity to the relevant acoustic cues for fricatives, we can entertain three possible outcomes. First, although aspiration duration and frication noise duration cues are used in both English and Korean fricative contrasts, it is possible that the boundary for Korean fricative contrast (analogous to the +30ms VOT range for stops) may be more salient than that for English fricatives (analogous to the 0ms VOT range for stops). In this case, Korean infants’ ability to discriminate the [s*]-[s] contrast might emerge earlier than English-learning infants’ discrimination of the [s]-[z] pair. Alternatively, we can also speculate that a cross-linguistically rare fricative contrast [s*]-[s] is harder to discriminate than the [s]-[z] contrast, which occurs more commonly in the world’s languages. This may be analogous to the rare nasal contrast [n]-[ŋ] in Filipino language (Tagalog) being more difficult for young Filipino infants to discriminate than the [m]-[n] contrast, which occurs more widely [12]. This would predict that Korean infants’ discrimination of the [s*]-[s] contrast would appear later than English-learning infants’ discrimination of [s]-[z]. Finally, it is also possible that Korean infants’ discrimination of the [s*]-[s] pair emerges at around the same time as English infants’ discrimination of the [s]-[z] contrast, which would indicate that fricative voicing contrasts are generally difficult for younger infants, and that this difficulty transcends the rarity of the Korean [s*]-[s] contrast.

## Methods

### Participants

Twenty-one 4- to 6-month-old infants (7 girls, mean age: 165.19 days, age range: 138‒209 days) and 23 7- to 9-month-olds (9 girls, mean age: 254.79 days, age range: 215‒301 days) participated in this experiment. All infants were born full term and healthy according to parental reports. An additional 19 infants were tested but excluded from the final analysis for the following reasons: crying (*n* = 8), parental intervention (*n* = 2), experimenter error (*n* = 8), and failing to habituate during 28 habituation trials (*n* = 1). Their parents gave written informed consent before the experiment. This study was approved by the Chung-Ang University IRB.

### Stimuli

Ten naturally uttered tokens of lenis /sa/ and fortis /s*a/ (20 tokens total) were used, recorded by a female Korean native speaker (first author) in Infant-Directed Speech (IDS) style (using a Marantz PMD 661 recorder). Paired t-tests were conducted to confirm the acoustic differences between the two sound groups. Acoustic characteristics of the stimuli and the results of acoustic analyses of the tokens are summarized in Table 1.

**Table 1:** Acoustic characteristics of Korean lenis versus fortis fricatives.

Acoustic parameter	Lenis /sa/		Fortis /s*a/		*t*
	M	SD	M	SD	
Duration (msec)	600	33	618	32	-1.152
Intensity (dB)	69.919	1.421	69.841	1.25	0.237
F0 (Hz)	311.568	46.410	323.453	40.060	-0.723
Aspiration duration (msec)	70.4	19	3.6	1	10.963**
Noise duration (msec)	123.27	10	170.07	30	-3.940*
H1-H2 (dB)	12.340	4.458	-4.510	5.794	6.232**
Centroid frequency (Hz)	8552.970	655.629	9183.966	378.229	-2.876*

* *p* < .05.

** *p* < .001.

The acoustic properties of all sound stimuli were analyzed in Praat by a well-trained researcher (first author). The noise duration [24] was measured from the onset of noise to the onset of a distributed spectrum characteristic of aspiration. The aspiration duration was measured between the end of the noise duration and the onset of the vowel duration (i.e., [24]). The total duration was measured from the onset of the noise duration to the end of the vowel duration. Centroid frequency was measured over an average spectrum of the middle 50ms of the noise duration. H1-H2 was measured as the amplitude difference between the first and second harmonics during the first 25ms of the vowel [24]. Measurements of f0 onset were taken by converting the average wavelength of the first 25ms of the vowels. Intensity was measured using the average of intensity during the vowel.

**Fig 1:**
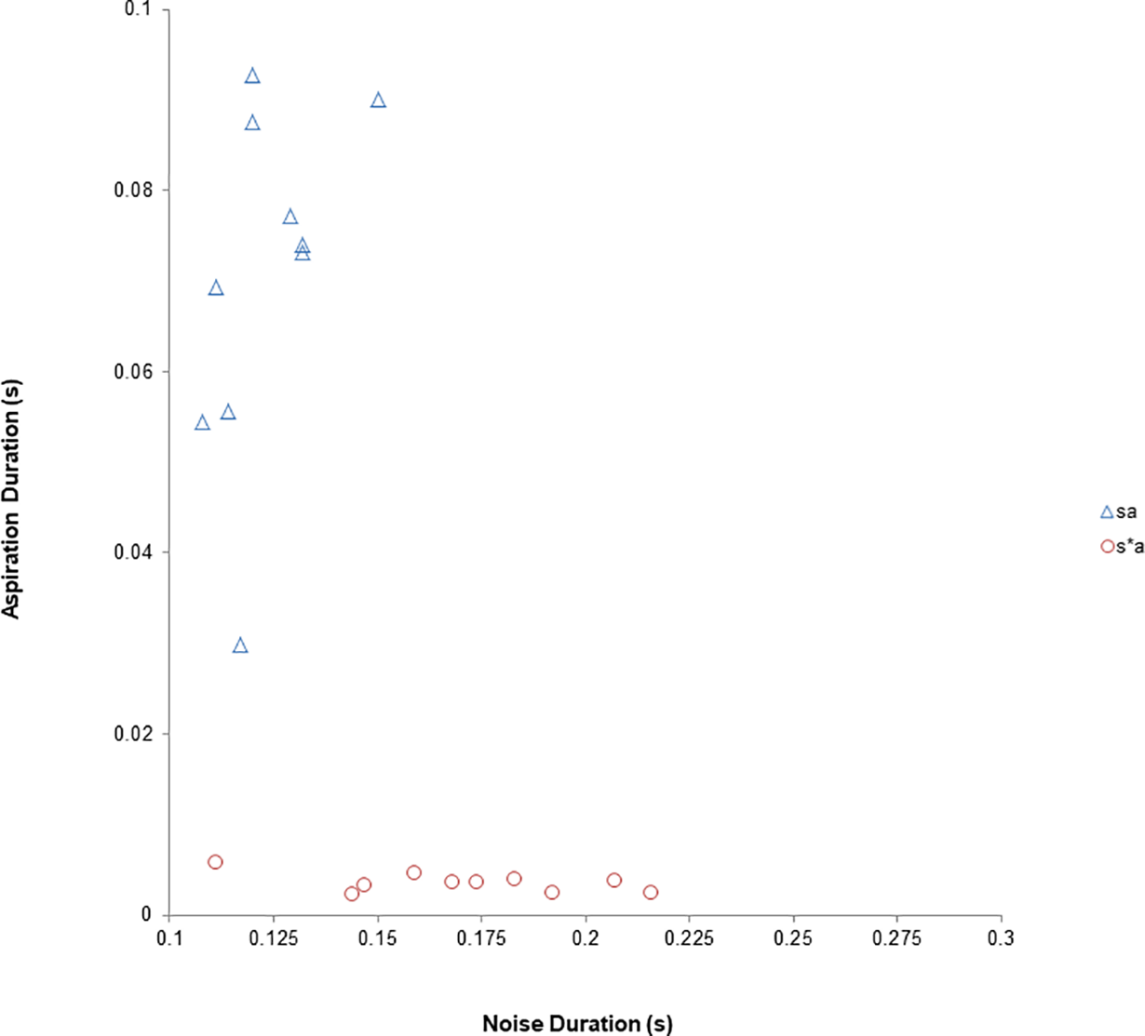
Distribution of lenis /sa/ and fortis /s*a/ tokens as a function of aspiration and frication noise duration used in the study. Circles represent fortis fricatives; triangles represent lenis fricatives. The data represent tokens naturally uttered by a female native Korean speaker in IDS style.

As mentioned above, adult Korean listeners rely strongly on frication noise duration to distinguish lenis from fortis fricatives [24, 25]. Aspiration duration (*M*_sa_ = 70.4, *SD* = 19 and *M*_s*a_ = 3.6, *SD* = 1; *t*(9) = 10.963, *p* < .001) and noise duration (*M*_sa_ = 123.27, *SD* = 10 and *M*_s*a_ = 170.07, *SD* = 30; *t*(9) = −3.940, *p* = .003) were significantly different between the two sound groups. Lenis /sa/ had a longer aspiration duration than fortis /s*a/, but fortis /s*a/ had longer frication noise than lenis overall. The two sound categories also differed by two additional cues: (1) H1−H2, which indicates amplitude differences between the first and second harmonics, with the lenis having a larger value than the fortis group, and (2) centroid frequency, which shows higher frequency for fortis than lenis. These characteristics were very similar to those reported earlier [28]. However, differences were not found in overall syllable duration (*M*_sa_ = .600, *SD* = .033 and *M*_s*a_ = .62, *SD* = .03; *t*(9) = −1.152, *p* = .279), intensity (*M*_sa_ = 69.919, *SD* = 1.421 and *M*_s*a_ = 69.841, *SD* = 1.25; *t*(9) = 0.237, *p* = .818), or f0 (*M*_sa_ = 311.568, *SD* = 46.409 and *M*_s*a_ = 323.453, *SD* = 40.059; *t*(9) = −.723 *p =* .488). Fig 1 further shows that the two sound categories are well separated by aspiration and frication duration.

Before testing infants, we first checked the discriminability of our stimuli with native adult listeners of Korean. Twenty Korean adults (mean age = 22.85 years, range = 20 to 27 years, female = 10) were asked to identify the sound after hearing the sample by clicking on the appropriate syllable choice from the two choices displayed on the screen [23–25]. Each stimulus was presented once, and the order of presentation was randomized. Overall, Korean adult listeners were highly accurate in identifying the sound categories (range of accuracy: 95% to 100% (*M* = 98.35, *SD* = 1.424), all of them significantly higher than chance level (50% chance with two syllable choices), *t*s(19) > 15.666, *p*s *<* .001).

For infant experiments, four lists of each stimulus group were prepared by randomly sequencing 10 tokens of each sound group, resulting in a total of eight playlists. Stimulus onset asynchrony was made about 1.5 seconds between tokens and thus the length of each playlist was approximately 15 seconds in total. Also, several tokens of naturally spoken /panta/ were recorded by the same female Korean native speaker to present during the pretest and posttest trials.

### Procedure

Infant testing was conducted in a dimly-lit sound-attenuated room controlled by E-Prime 2.0 software (Psychology Software Tools, Pittsburgh, PA) [32] on a laptop computer located in an adjacent room. The infants sat on their parents’ laps facing a monitor, which was placed 1 meter (approximately 39 inches) away. Two speakers, placed 40 cm (approximately 15 inches) away from each other, were located below the monitor, from which the stimuli were presented at approximately 60−65dB sound pressure level. An experimenter monitored the infants’ visual responses in the adjacent control room via a video camera (Sony HDR-CX700) and the responses were simultaneously recorded by a recorder (EzRecorder 130) for later offline coding. Both the experimenter and the parent wore headphones (Sony MDR-7506) to mask the auditory stimuli presented to the infants.

A modified version of the visual habituation method [10–11, 30] was used to test infants’ discrimination of the sound pair, in which increased looking time after a sound change after habituation to one sound category indicates discrimination.

The infants were first presented with a pretest trial, hearing the sound /panta/ while watching an animated display (i.e., a ladybug flying around). The habituation phase then followed. During the habituation phase, one of the four lists from either of the two sound categories was randomly played, together with a red and black checkerboard image on the screen. As soon as the infant turned towards the monitor, the experimenter pressed the key on the computer to enable the control software to measure and calculate the cumulative looking time. The habituation phase ended if the average looking time on the last four habituation trials fell below 65% of the average looking time during the first four habituation trials, or if a maximum of 28 habituation trials was reached. Approximately half of the infants were habituated to one sound category in each condition.

Immediately after the habituation phase ended, the test phase began, consisted of two trials, the Switch (different sound category from habituation) and Same (same sound category as habituation) trials. Half of the infants performed the same trial first followed by the switch trial, and the remaining half performed the switch trial before the same trial. Each trial began as soon as the infant successfully oriented toward the monitor. During each test trial, a red and black checkerboard image was displayed on the screen together with the target sound (the same or different). An animation portraying a brightly colored moving chick was played to grab the infant’s attention. Each trial lasted about 15 seconds, the same as the length of each playlist (1.5 sec x 10 tokens). The experiment ended with a posttest trial (the same as the pretest trial) to check whether the infant had become tired/uninterested during the test phase.

Two trained coders, blind to the trial information, manually coded infants’ looking times during the test trials by viewing the recorded video frame by frame (29.97 frames per second). Two coders fully double-coded 30% of the data and their coding agreement was 99.01%. Looking times during the pretest and posttest trials were obtained from online computer key press data.

## Results

First, we checked whether infants were able to maintain interest throughout the experiment by comparing the looking times measured during the pretest and posttest trials. The mean looking times during the posttest trials did not significantly decrease for either group: 4‒6-month-olds (*M*_pretest_ = 13.752s, *SD* = 2.377s, *M*_posttest_ = 14.290s, *SD* = 1.371s, 95% confidence interval (CI) of the difference [−1.319, 0.234], *t*(20) = −1.452, *p* = .162), and 7‒9-month-olds (*M*_pretest_ = 14.135, *SD* = 1.279, *M*_posttest_ = 14.262, *SD* = 1.833, 95% CI of the difference, [−.960 to .706], *t*(22) = −.316, *p* = .755). These results indicate that both groups of infants were able to maintain interest and attention until the end of the experiment.

**Fig 2:**
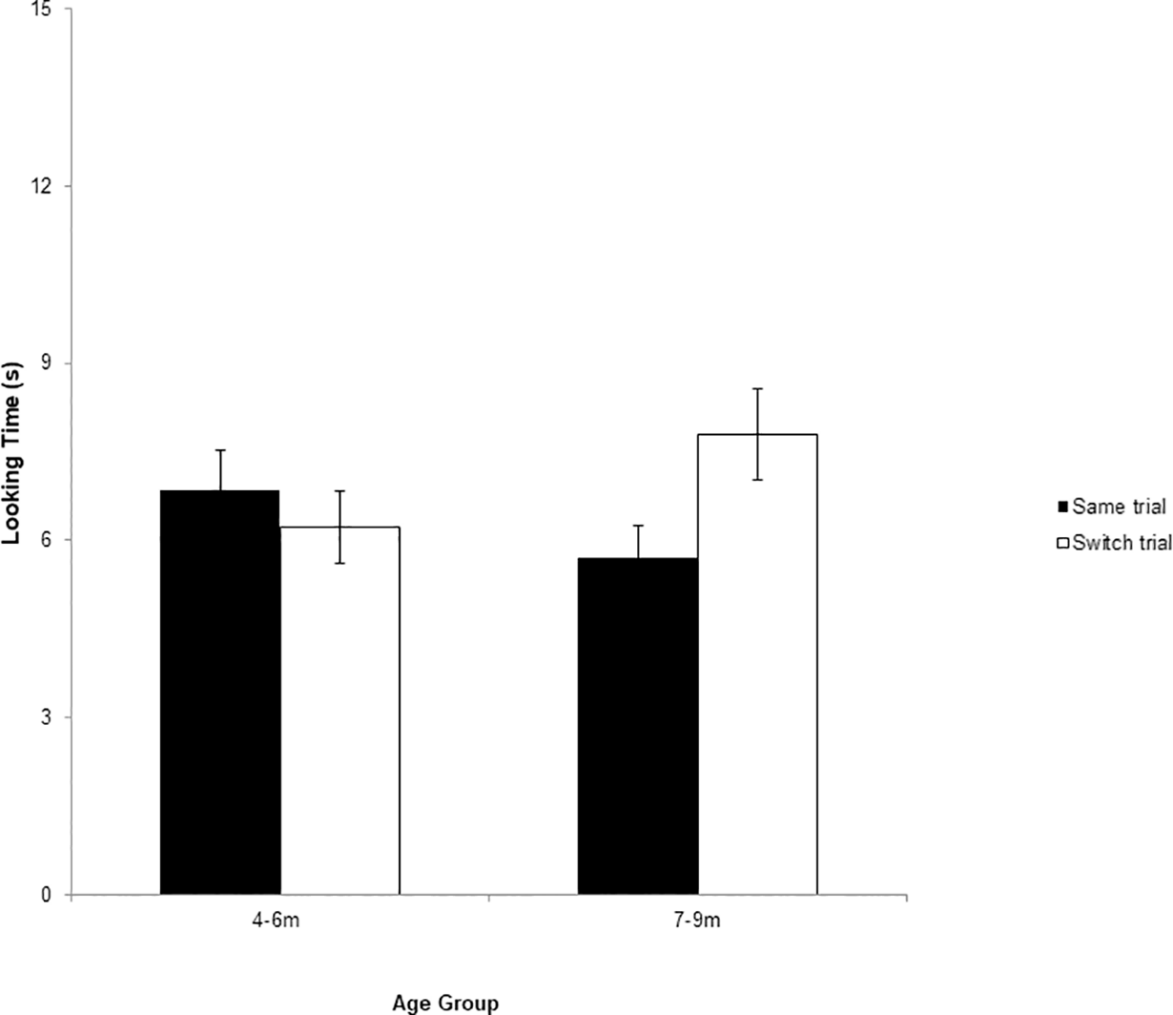
Mean looking times during the same and switch trials for naturally uttered lenis /sa/ and fortis /s*a/ sounds among 4- to 6-month-olds and 7- to 9-month-olds. The 7- to 9-month-olds exhibited significantly longer looking times between the switch trials than the same trials. * *p* < .05. Error bars indicate standard errors of the means.

The average looking times for the same and switch trials were calculated and submitted to a 2 (age group) x 2 (habituation stimuli) x 2 (trial type) repeated-measures analysis of variance (ANOVA), with age (4- to 6-months, 7- to 9-months) and habituation stimuli (/sa/, /s*a/) as between-participant variables and the trial type (same, switch) as a within-participant variable. The results show that none of the main effects were significant: for trial (*F*(1, 40) = 1.792, *p* = .188, partial *η* ^2^ = .043), age (*F*(1, 40) = .071, *p* = .791, partial *η* ^2^ = .002), and habituation stimuli (*F*(1, 40) = 1.521, *p* = .225, partial *η* ^2^ = .037). However, a significant interaction effect was observed between the age group and trial type, *F*(1, 40) = 6.171, *p* = .017, partial *η* ^2^ = .134. As shown in Fig 2, 7- to 9-month-old infants looked longer during the switch trials than the same trials, while the looking times were not significantly different for 4- to 6-month-olds. Other interaction effects were not found to be significant (*F*s < 2.95, *p*s > .09).

To further examine the effect of the trial type and habituation stimuli for each age group, two-way repeated-measures ANOVA were performed separately. For the older group, trial type had a significant effect (*F*(1, 21) = 6.807, *p* = .016, partial *η* ^2^ = .245, *M*_same_ = 5.706, *SD*_same_ = 2.586, *M*_switch_ = 7.792, *SD*_switch_ = 3.683). An interaction between trial type and habituation stimuli was marginally significant (*F*(1, 21) = 3.807, *p* = .065, partial *η* ^2^ = .153), indicating a tendency for a directionality effect [33]. When the infants were habituated to the /s*a/ sound, their looking time differences between the switch and same trials tended to be larger than for those who were habituated to the /sa/ sound (Mean difference in /s*a/ habituation group = 3.531, *SD* = 4.591, 95% CI of the difference = [0.614, 6.448]; Mean difference in /sa/ habituation group = 0.509, *SD* = 2.392, 95% CI of the difference = [−1.097, 2.117]). Though this was only a tendency, it may be indicative that the fortis category might be used as an anchor for categorical discrimination, similar to prior findings on the asymmetrical pattern of vowel category perception [11].

For the younger group, neither the trial type (*F*(1, 19) = 0.728, *p* = .404, partial *η* ^2^ = .037) nor the interaction between the trial type and the habituation stimuli (*F*(1, 21) = 0.181, *p* = .676, partial *η* ^2^ = .009) were significant. These results show that 4‒6-month-olds failed to discriminate the fricative pair regardless of the type of sound they were habituated to (*M*_same_ = 6.838, *SD*_same_ = 3.123, *M*_switch_ = 6.218, *SD*_switch_ = 2.780; 95% CI of the difference = [−.828, 2.067]).

Of the 21 4−6-month-old infants tested, 10 (47.6%) showed longer looking times during the switch trials than the same trials, whereas the others showed longer looking times during the same trials. However, 16 of the 23 (69.6%) 7−9-month-olds showed longer looking times during the switch trials, whereas the remainder of the infants showed longer looking times during the same trials. These patterns show that, consistent with the average duration of looking time, 7−9-month-olds discriminated the lenis and fortis fricative contrast, but 4−6-month-olds did not.

## Discussion and conclusion

In this study, we predicted that Korean infants’ discrimination of fricative contrast would emerge in the enhancement pattern, based on previous studies in English. Our findings confirmed this prediction, and showed that Korean infants’ discrimination of the [s*a]-[sa] contrast does emerge following the enhancement pattern. This adds to the growing evidence [9–13, 17, 18] that there are native phonemic contrasts that infants cannot discriminate at a young age but become able to discriminate as they grow older. As discussed in the introduction, these results could elucidate the developmental changes that make it possible for older infants to discriminate these contrasts.

Regarding the timing of when Korean infants’ discrimination ability emerges, we considered three alternative hypotheses: 1) Korean infants’ discrimination emerges earlier than that reported in English-learning infants, 2) Korean infants’ discrimination emerges later than English-learning infants’, and 3) they emerge at around the same time. We found that Korean infants’ ability to discriminate [s*]-[s] contrast emerged at 7‒9 months of age, which is a similar timeline to that of English-learning infants’ discrimination of the /s/-/z/ fricative contrast [21], and consistent with our third hypothesis.

Note that this is relatively early timing compared to other studies that have reported the enhancement pattern of development, many of which examine rare contrasts among the world’s languages. The Filipino /n/-/ŋ/ nasal contrast discrimination ability was reported to emerge after 10 months [12], Japanese phonemic vowel duration (e.g., long versus short vowels) [11] and geminate obstruent [10] discrimination abilities were observed after around 9 months, and the English /d/-/ð/ discrimination after 12 months [9].

As illustrated earlier, the Korean fricative contrast /s*/-/s/, for which both fricatives are voiceless, is a rarely observed contrast among the languages of the world [23–26, 28, 29]. However, the fact that Korean infants became capable of discriminating this contrast with a timing similar to that seen in English indicates that the rarity of the contrast does not necessarily make it more difficult for infants to discriminate than the more common voiced-voiceless contrast (e.g., /z/-/s/). This is different from another rare contrast, the Filipino /n/-/ŋ/ nasal sounds, the discrimination of which does not emerge until 10‒12 months of age [12]. The late emergence of this contrast has been attributed to the low acoustic salience of this pair compared to the /m/-/n/ contrast, which is more common and higher in acoustic salience. While acoustic salience may play a role in the discrimination of some contrasts, the similarity in the age at which infants become able to discriminate the /s*/-/s/ and /s/-/z/ contrasts cannot be attributed to the acoustic salience of these sounds.

As discussed above, the key acoustic cues that are used to distinguish English /z/-/s/ are similar to those used in Korean fricative perception, although each language utilizes a different set of acoustic parameters for their unique identifications. Among other cues, frication noise duration has been reported to be key to discriminating both the Korean /s*/-/s/ and the English /z/-/s/ pairs [23–25, 29, 34]. Considering this similarity, we find it interesting that the onset of the ability to discriminate these fricative pairs was observed at similar developmental periods. This suggests the possibility that the development of infants’ ability to discriminate certain segments is related to their developing sensitivity to the specific type of acoustic parameters associated with each categorical identification/distinction. For instance, the ability to reliably apply frication noise duration to fricative sound discrimination may develop relatively slowly compared to other cues such as voicing [15, 16, 18, 19] or salient formant cues in nasal contrast discrimination as in /m/-/n/ [12].

Our data point to the idea that infants’ developing sensitivity to a diverse set of cues can play a major role in the development of native phonology. For instance, Japanese infants’ use of vowel duration cues at the phonemic level did not develop until 9 months of age [11]. When it comes to utilizing closure duration cues to discriminate geminate consonants in Japanese, Japanese infants took about 11 months to become able to do so unless there were redundant co-varying cues available to facilitate the perception of the younger infants (9.5 months) [10].

In a recent study, Choi and her colleagues [35] also showed that Korean infants’ ability to discriminate three-way stop contrasts emerged at different times depending on various acoustic cues that are associated with each contrast. For stop contrasts (i.e., fortis-aspirated pairs) that could be discriminated on the basis of the VOT cue, Korean infants could reliably distinguish them from 4 months of age. However, they could not discriminate the stop contrasts (i.e., lenis-aspirated pairs) that require them to integrate VOT cues and f0 cues until the age of 10‒12 months. Taken together with our current data, these findings point toward the possibility that developing sensitivity to different acoustic cues and abilities to utilize these cues may be responsible for determining which sound contrasts/categories emerge earlier or later.

Clearly, many more studies are needed to identify the specific acoustic parameters to which infants are sensitive at various developmental stages. For fricative discrimination, for example, it needs to be examined whether frication noise duration is the cue that Korean/English infants rely on, as reported in an early study with a small sample of English infants [21]. Aspiration duration is another cue that could play an important role in Korean fricative perception [24, 25]. Korean adults start perceiving the respective sound as /s/ as the aspiration duration becomes longer. Chang [24] also reported that f0 at the vowel onset following fricative consonants can contribute to Korean fricative discrimination, although its contribution was smaller than frication noise duration or aspiration duration in adults’ perception. Therefore, it is also possible that infants need to develop the ability to consider and utilize multiple cues (e.g., fricative noise duration, aspiration duration, f0, etc.) in identifying the fricative sounds based on their experience with the language.

In production, fricatives are the sounds known to take the longest before children can produce them correctly. In English-learning children’s production, fricatives are often replaced with other sounds (e.g., /s/ replaced with the stop /t/) early on. Among children with speech sound disorders in English, fricative sounds are known to be the hardest sounds to learn to pronounce. A similar difficulty in production has been observed in Korean children as well [36–38]. It is estimated that after going through periods in which they omit and substitute the fricative sounds early on, it takes Korean children 6‒7 years to master their production [36]. Although children’s production of segments develops at a much slower pace than their perception, it is interesting to note the parallel pattern between stops and fricatives. In general, infants have generally been found to discriminate stop contrasts from a very young age, while the discrimination of fricatives seems to emerge later. When mothers talk to infants in IDS, they tend to produce fricatives less frequently than in ADS in many languages, including English [39], Korean [40], and Japanese [41]. The dominant explanation of why this happens is that mothers adjust their speech to match young children’s production. It is an interesting question whether the less frequent occurrence of fricatives in their input is related to a slower development of infants’ discrimination of fricative contrasts.

The current data present an additional case in which infants cannot discriminate native sound pairs early in their development and need additional input to develop native phoneme categories. Our data, together with a growing body of other evidence, seem to point toward the idea that the early repertoires of phonemes that infants can discriminate might actually be somewhat limited, suggesting that earlier perceptual capacities are more limited than has been believed [1, 10–13, 35]. This calls for the need to test wider sets of contrasts used across the various languages of the world, and to test infants learning a wider range of languages that have not yet been tested. The current report on Korean infants’ development of a rare fricative contrast is the first step toward this goal of illuminating more precise nature of infants at earlier ages as their abilities develop during their development of native phonology.
